# The Role of Cannabinoids in the Setting of Cirrhosis

**DOI:** 10.3390/medicines5020052

**Published:** 2018-06-09

**Authors:** Pratima Dibba, Andrew A. Li, George Cholankeril, Umair Iqbal, Chiranjeevi Gadiparthi, Muhammad Ali Khan, Donghee Kim, Aijaz Ahmed

**Affiliations:** 1 Division of Gastroenterology, Women and Infants Hospital/Warren Alpert School of Medicine, Brown University, Providence, RI 02905, USA; pratima_dibba@brown.edu; 2 Department of Medicine, Stanford University School of Medicine, Stanford, CA 94304, USA; andrewli@stanford.edu; 3 Division of Gastroenterology and Hepatology, Stanford University School of Medicine, Stanford, CA 94304, USA; georgetc@stanford.edu (G.C.); dhkimmd@stanford.edu (D.K.); 4 Department of Medicine, Mary Imogene Bassett Hospital, Cooperstown, NY 13326, USA; umairiqbal_dmc@hotmail.com; 5 Division of Gastroenterology and Hepatology, University of Tennessee Health Science Center, Memphis, TN 38163, USA; chirudoc@yahoo.com (C.G.); mkhan24@uthsc.edu (M.A.K.)

**Keywords:** cirrhosis, treatment of cirrhosis, endocannabinoid system, endocannabinoids, exocannabinoids, THC, CB1, CB2, cannabinoids

## Abstract

Although the mortality rates of cirrhosis are underestimated, its socioeconomic burden has demonstrated a significant global impact. Cirrhosis is defined by the disruption of normal liver architecture after years of chronic insult by different etiologies. Treatment modalities are recommended primarily in decompensated cirrhosis and specifically tailored to the different manifestations of hepatic decompensation. Antifibrogenic therapies are within an active area of investigation. The endocannabinoid system has been shown to play a role in liver disease, and cirrhosis specifically, with intriguing possible therapeutic benefits. The endocannabinoid system comprises cannabinoid receptors 1 (CB1) and cannabinoid receptor 2 (CB2) and their ligands, endocannabinoids and exocannabinoids. CB1 activation enhances fibrogenesis, whereas CB2 activation counteracts progression to fibrosis. Conversely, deletion of CB1 is associated with an improvement of hepatic fibrosis and steatosis, and deletion of CB2 results in increased collagen deposition, steatosis, and enhanced inflammation. CB1 antagonism has also demonstrated vascular effects in patients with cirrhosis, causing an increase in arterial pressure and vascular resistance as well as a decrease in mesenteric blood flow and portal pressure, thereby preventing ascites. In mice with hepatic encephalopathy, CB1 blockade and activation of CB2 demonstrated improved neurologic score and cognitive function. Endocannabinoids, themselves also have mechanistic roles in cirrhosis. Arachidonoyl ethanolamide (AEA) exhibits antifibrogenic properties by inhibition of HSC proliferation and induction of necrotic death. AEA induces mesenteric vasodilation and hypotension via CB1 induction. 2-arachidonoyl glycerol (2-AG) is a fibrogenic mediator independent of CB receptors, but in higher doses induces apoptosis of HSCs, which may actually show antifibrotic properties. 2-AG has also demonstrated growth-inhibitory and cytotoxic effects. The exocannabinoid, THC, suppresses proliferation of hepatic myofibroblasts and stellate cells and induces apoptosis, which may reveal antifibrotic and hepatoprotective mechanisms. Thus, several components of the endocannabinoid system have therapeutic potential in cirrhosis.

## 1. Introduction

Cirrhosis results from chronic insults to the liver, causing persistent wound healing and fibrosis that result in the disruption of normal liver architecture [1]. The socioeconomic burden is vast [2] and mortality rates, although underestimated globally, are high [3,4]. Multiple etiologies can cause liver injury, all of which risk progression to cirrhosis [1]. The transition of liver parenchyma from initial injury to cirrhosis is scored by the MATEVIR scoring system based on histologic progression [5]. Treatment recommendations are focused on specific interventions for manifestations of decompensated cirrhosis such as ascites, spontaneous bacterial peritonitis, hepatic encephalopathy, and variceal hemorrhage. However, antifibrogenic treatment modalities are still being studied. The endocannabinoid system and its pharmacomodulation have shown promising therapeutic benefits in cirrhosis. Because of the socioeconomic burden of cirrhosis, partially attributed to the lack of antifibrogenic therapies, and the concurrent increased use of recreational and therapeutic marijuana, the investigation of endocannabinoid system in cirrhosis may be beneficial based on its pro- and anti-fibrogenic properties.

## 2. Epidemiology

Approximately 3.9 million adults in the United States were diagnosed with liver disease in 2015 [6]. A study conducted the same year estimated the prevalence of cirrhosis as 0.27%, with a higher prevalence in non-Hispanic blacks and Mexican Americans, those living below the poverty level, and those with an education level less than high school [3]. Death rates for chronic liver disease and cirrhosis within the United States increased 31% between 2000 and 2015 in both men and women between ages 45 and 64 [3]. In 2004, the direct costs of chronic liver disease and cirrhosis in the United States (excluding patients with hepatitis C) were estimated at $2.5 billion, and indirect costs were estimated at $10.6 billion [2]. The societal impact of cirrhosis includes total costs and reduced employment, especially in those who have received liver transplants [2]. Although the global health burden of cirrhosis is undeniable, the global mortality data is vague due to a paucity of information in 58 out of 187 countries, primarily in Africa [4].

## 3. Definition, Etiologies and Clinical Presentation

Cirrhosis is defined as a condition that disrupts the normal architecture of the liver. It is caused by chronic insults which lead to persistent wound healing and hepatic parenchymal fibrosis resulting in progressive, diffuse, fibrosing architecture [1]. Common causes include alcohol, biliary obstruction, biliary cirrhosis, chronic hepatitis B or C, hemochromatosis, and NAFLD [1]. Less common causes include autoimmune hepatitis, drugs/toxins, genetic metabolic diseases, infection, vascular abnormalities, veno-occlusive disease, and idiopathic etiologies. In early and compensated disease, patients can present with anorexia, weight loss, fatigue, weakness, and osteoporosis [1]. In decompensated disease, patients may present with ascites, variceal bleeding from portal hypertension, hepatic encephalopathy, spontaneous bacterial peritonitis, jaundice, icterus, pruritus, or coagulopathy [1]. 

Physical exam findings may additionally reveal spider angiomata, spider telangiectasias, gynecomastia, fetor hepaticus, clubbing, hypertrophic osteoarthropathy, Cruveilhier–Baumgarten murmur, Dupuytren’s contracture, Kayser–Fleischer rings, nail changes, palmar erythema, splenomegaly, or testicular atrophy [1].

## 4. Pathogenesis

Liver fibrosis occurs as a result of exposure to chronic stimuli (i.e., aforementioned etiologies) that cause insult to its architecture [7]. Chronic stimuli cause progressive accumulation and decreased remodeling of the extracellular matrix which can result in fibrosis and progression to cirrhosis [7]. The extracellular matrix transitions from a normal low-density basement-membrane type matrix to an interstitial type, which influences hepatocytes, hepatic stellate cells (HSCs), and endothelial cells [8]. Activation of HSCs is a primary event in hepatic fibrosis [9]. HSCs, found in the space of Disse storing vitamin A and retinoids, are activated by inflammatory cytokines such as platelet-derived growth factor, transforming growth factor-beta (TGF-β), tumor necrosis factor-α, and interleukin-1 [9]. Their activation results in collagen and extracellular matrix deposition as well as their transformation into myofibroblasts [9]. Additionally, defenestration of liver sinusoidal endothelial cells and their subsequent capillarization contribute to hepatocyte dysfunction [9]. Kupffer cells, which are activated by injurious stimuli, also mediate inflammation, further stimulating injury and fibrosis [9]. Apoptosis of the parenchymal cells of the liver (hepatocytes) promotes inflammation, fibrogenesis, and development of cirrhosis due to the release of reactive oxygen species and fibrogenic mediators, activation of HSCs, and stimulation of myofibroblasts [9]. Hypoxic hepatocytes secrete TGF-β, which exacerbates fibrogenesis [9]. Additionally, the extracellular matrix can amplify fibrosis [7] via changes in membrane receptors that oppose focal adhesion, activation of matrix matalloproteases to release fibrogenic and proliferative growth factors, and stiffening of the matrix [10].

Cytokines and microRNAs also play major roles in mediating cirrhosis [7,9]. Cytokines are predominantly produced by CD4 T-helper lymphocytes [7]. Platelet-derived growth factor activates HSCs and reduces extracellular matrix degradation, resulting in mitogenic and fibrogenic effects [9]. TGF-β is pro-fibrogenic as it inhibits degradation of extracellular matrix, and causes deposition of collagen [9]. It also induces apoptosis of hepatocytes by inhibiting DNA synthesis [9]. Tumor necrosis factor-α activates HSCs and increases synthesis of extracellular matrix [9]. Interleukins, some of which are anti-fibrogenic, can also be pro-fibrogenic [9]. Interleukin-1 activates HSCs and promotes their production of matrix metalloproteinases, which causes fibrosis [9]. Interleukins 17, 22, and 6 have also been named pro-inflammatory and pro-fibrogenic [9]. Micro RNAs control fibrosis progression and are expressed by HSCs [7]. Specific pathogenic mechanisms can also be associated with type of injury, such as alcohol-induced injury and viral hepatitis [7].

## 5. Diagnosis

The gold standard for the diagnosis of cirrhosis and the underlying etiology for liver injury is a liver biopsy [5]. Liver biopsy yields a sample of core liver tissue that is sent for pathologic analysis [5]. Degree of fibrosis is then scaled by different scoring systems including the Ishak, METAVIR, Scheuer, and Batts-Ludwig scoring systems [5]. The diagnostic accuracy of the biopsy, however, can be affected by the size of the sample, etiology of the liver disease, and inter-observer variability [5]. Complications of liver biopsy include mortality from hemoperitoneum, specifically in those with hepatocellular carcinoma or cirrhosis, hypotension, or post-procedural pain [5]. The American Association of the Study of Liver Diseases (AASLD) recommends that liver biopsy be obtained when the diagnosis is unclear or when a specific diagnosis may alter management and can be considered if fibrosis stage is necessary to guide treatment [11].

Therefore, due to the liver biopsy’s invasive nature, limitations, and potential for complications, non-invasive assessments are being increasingly utilized and recommended. These include radiologic techniques, elastography techniques, and indirect and direct serum biomarkers [5]. Radiologic techniques include ultrasound, computed tomography, and magnetic resonance imaging [5]. Elastography techniques include transient elastography (i.e., Fibroscan), acoustic radiation force imaging, supersonic shear wave imaging, and magnetic resonance elastography [5]. Indirect serum biomarkers include the Aspartate Aminotransferase-Platelet Ratio Index (APRI), fibrotest (also known as Fibrosure), FIB4 (which is calculated using age, aspartate aminotransaminase, platelet count, and alanine transaminase), Nonalcoholic Fatty Liver Disease (NAFLD) Fibrosis score, and fibroindex [5]. Direct markers include hyaluronic acid, amino terminal of serum procollagen III peptide (PIIINP), chondrex (YKL-40), and the ELF score [5].

## 6. Classification

Cirrhosis can be subcategorized into compensated and decompensated cirrhosis based on prognostic stage [12]. Patients can further be risk stratified by their Child-Turcotte-Pugh classification [12]. This scoring system is based upon serum bilirubin, serum albumin, prothrombin time, ascites, and grade of encephalopathy [13]. Compensated cirrhosis is generally asymptomatic but can be further categorized into those with mild portal hypertension versus those with clinically significant portal hypertension, in whom the hepatic venous pressure gradient is greater than 10 [12]. Those with clinically significant disease are at risk of complications including ascites, encephalopathy, varices, variceal hemorrhage, postsurgical decompensation and hepatocellular carcinoma [12]. Serum albumin, presence of gastroesophageal varices, and Model for End-Stage Liver Disease [MELD] are predictors of decompensation in these patients [12]. Decompensated cirrhosis refers to those who possess one of these complications in the setting of cirrhosis [12]. This classification is specifically relevant to treatment guidelines posed by the AASLD in terms of portal hypertensive bleeding [12]. The MELD score is also used in determining prognosis in cirrhosis patients as well as in the allocation of donor organs for liver transplantation [13].

## 7. Natural History

The METAVIR score stages fibrosis based on histologic progression [5]. Stage F0 indicates no fibrosis; F1 indicates portal fibrosis without septa; F2 indicates presence of few septa; F3 indicates numerous septa without cirrhosis; and F4 indicates cirrhosis [5]. Cirrhosis can remain compensated for years and can transition to decompensated cirrhosis at a rate of 5–7% per year [13]. Median survival rate for compensated cirrhotic patients ranges between 9 and 12 years [13]. Decompensated cirrhotic patients have poor survival with a 1-year survival rate less than 50% in patients with ascites and variceal hemorrhage [13].

## 8. Treatment/Management

The treatment of cirrhosis largely involves specific management of complications of cirrhosis, treatment of etiologies to prevent development of cirrhosis, and liver transplantation based on etiology of cirrhosis [14]. For those with ascites, first-line treatment as recommended by the AASLD includes cessation of alcohol use if present, sodium restriction, diuretic therapy, discontinuation of non-steroidal anti-inflammatories, and evaluation for liver transplant [15]. Second-line therapy as recommended by the AASLD includes discontinuation of beta blockers, angiotensin converting enzymes, and angiotensin receptor blockers, consideration of midodrine, therapeutic paracenteses, evaluation for liver transplantation, and/or transjugular intrahepatic portosystemic shunt (TIPS) [15]. Third-line treatment as recommended by the AASLD includes a peritoneovenous shunt [15]. The European Association for the Study of Liver (EASL) recommends no intervention for mild ascites, restriction of sodium intake and diuretics for moderate ascites evident by symmetrical distention of the abdomen, and large volume paracentesis followed by sodium restriction and diuretics in those with large or gross ascites [16]. Therapies proposed by EASL for refractory ascites include repeated large volume paracenteses, TIPS under certain criteria, and peritovenous shunt [16].

For those patients with spontaneous bacterial peritonitis (SBP), treatment includes the use of antibiotics as recommended by EASL and AASLD with chemoprevention thereafter to prevent recurrence [15,16]. Recommendations regarding management of hyponatremia include fluid restriction and use of vaptans in certain scenarios [15,16]. If a patient develops hepatorenal syndrome, EASL suggests specific monitoring parameters and potentially, drug therapy and renal replacement therapy; ultimately, EASL recommends liver transplantation [16]. AASLD also recommends evaluation for liver transplantation for those with hepatorenal syndrome [15]. For those who develop hepatic encephalopathy, lactulose is the recommended first-line agent as suggested by AASLD and EASL [15]. Rifaximin is suggested to be added on to lactulose to prevent recurrence [15]. Guidelines also exist in the management of portal hypertensive bleeding secondary to cirrhosis based on very specific classifications and parameters [12].

In addition to management of complications of cirrhosis, other modalities of treatment associated with cirrhosis include treatment of the etiologies and diseases underlying cirrhosis [9]. Drug therapy in viral hepatitis, abstinence from alcohol, control of metabolic dysfunction, and chelation in the setting of copper and iron overload have been used as specific therapies for certain etiologies for liver disease [9]. Therapy targeting the pathogenesis of cirrhosis to treat the disease, itself, is still being studied. Anti-inflammatory and anti-oxidative drugs such as celcoxib, taurine, and vitamin E have shown antifibrotic effects [9]. Glucocorticoids, azathioprine, colchicine, and rapamycin have exhibited anti-inflammatory, anti-fibrotic, and immunomodulatory effects [9]. Drugs that have exhibited a hepatoprotective effect include silymarin, ursodeoxycholic acid, tauroursodeoxycholic acid, and verapamil [9]. Gene therapy, specifically miRNA-based therapy, has also been implicated as a therapeutic modality in cirrhosis [9]. Antifibrotic drug therapies, however, are still unavailable, although the endocannabinoid system is being studied as a target for pharmaco-modulation in the setting of cirrhosis.

## 9. The Cannabinoid System in Liver Disease

Cannabinoid receptors 1 (CB1) and cannabinoid receptor 2 (CB2) are two G-protein receptors identified within the endocannabinoid system that may play a role in liver disease [17]. CB1 and CB2 are activated by highly lipophilic ligands called endocannabinoids [17]. CB1 is predominantly found in the brain and is accountable for the psychotropic and behavioral effects manifested by cannabinoid use [17]. CB2 is expressed in the peripheral tissues functioning in the modulation of innate immunity and bone mass with some antitumor properties [17]. Within the liver, CB1 has been detected in endothelial cells and hepatocytes whereas CB2 has been detected in Kupffer cells and is prominently expressed in the cirrhotic liver [17,18]. Known endocannabinoids include anandamide (arachidonoyl ethanolamide, AEA) and 2-arachidonoyl glycerol (2-AG), which activate CB1 and CB2 [17]. Exocannabinoids, major components of cannabis, include tetrahydrocannabinol (THC), tetrahydrocannabivarin (THCV), and cannabidiol (CBD) [19,20]. THC acts on CB1, CB2, transient receptor potential cation channel subfamily C member 1, and G protein-coupled receptors GPR56 and GPR11 [19]. THCV is an analog of THC and function as a CB1/CB2 agonist in high doses and a CB1/CB2 neutral antagonist in lowdoses [21]. CBD functions as an indirect agonist on CB1 via increase of endocannabinoid tone or CB1 constitutional activity [21].

## 10. Cannabinoids in Cirrhosis

CB1 and CB2 agonism and antagonism have been studied in cirrhosis. Mallat et al. demonstrated that CB1 and CB2 are upregulated in cirrhotic liver samples [17]. CB1 activation enhances fibrogenesis whereas CB2 activation counteracts progression to fibrosis [17]. Conversely, in rodent models, deletion of CB1 demonstrated improvement of hepatic fibrosis and steatosis, and deletion of CB2 demonstrated increased collagen deposition, steatosis, and enhanced inflammation [19,22]. CB1 antagonism and/or CB1 deficiency not only reduces liver fibrosis, but has been shown to decrease TGF-β, increase apoptosis of activated myofibroblasts, and decrease activation of HSCs [23,24]. 

Studies prior to these findings showed that CB2 deficiency resulted in prolonged survival of liver fibrogenic cells, increasing fibrosis with increased deposition of fibrotic tissue [24,25]. In one study, CB2 antagonism in mice exposed to carbon tetrachloride exhibited enhanced fibrosis whereas stimulation of CB2 demonstrated prevention of fibrosis progression [24,26]. Mallat et al. also demonstrated that CB2 accelerates the regenerative response following acute liver injury, possibly related to paracrine effects of hepatic myofibroblasts [17]. Additionally, Julien et al. demonstrated that CB2 receptor activation exhibits antifibrogenic activity by triggering growth inhibition and apoptosis with a possible role in the counteracting liver fibrogenesis [25]. JWH-133, a CB2 selective agonist, was found to enhance the regenerative response to acute liver injury and accelerate liver regeneration [18]. Subsequent studies supported these findings with administration of JWH-133 resulting in improved liver fibrosis, decrease in inflammatory infiltrate, and decreased density of hepatic myofibroblasts secondary to apoptosis [27]. 

Mallat et al. demonstrated that CB1-deficient mice exhibited reduced fibrosis after exposure to carbon tetrachloride [26]. CB1 blockade or inactivation has been associated with reduced progression to fibrosis, reduced hepatic expression of TGF-β1, and decreased quantity of fibrogenic cells, thought to be secondary to antiproliferative and apoptotic properties of CB1 antagonism in hepatic cells [17]. In fact, in common bile-duct-ligated rodents with biliary cirrhosis, CB1 blockade in the hepatic microcirculation resulted in decreased collagen deposition in cirrhotic livers [28]. CB1 has also been implicated in liver regeneration, as CB1 blockade by rinomabant (SR141716A) or lack of CB1 receptors demonstrated reduced liver regeneration [29].

CB1 antagonism by rimonabant has also demonstrated effects on vasculature. It specifically causes increased arterial pressure with reversal of arterial hypotension, increased splanchnic vascular resistance, and reduced mesenteric blood flow and portal pressure in rodents with cirrhosis [19,24,30]. Rimonabant, therefore, demonstrated prevention of ascites [24,31,32].

Endocannabinoids, themselves, also have mechanistic roles in cirrhosis. AEA has exhibited antifibrogenic properties by inhibition of HSC proliferation and induction of necrotic death [30]. Like CB1 mechanisms, endocannabinoids may also cause hemodynamic alterations in cirrhosis by acting on CB1 on the splanchnic and hepatic vascular endothelium [33]. They may be involved in apoptosis of hepatocytes [33]. In rodents with cirrhosis, AEA was found to induce mesenteric vasodilation and hypotension via CB1 induction [18,19]. AEA was also found to contribute to cirrhotic cardiomyopathy as mediated by activation of CB1 receptors by lowering cardiac contractility [24]. AEA notably demonstrated induction of hepatocyte proliferation [19]. 2-AG was found to be a fibrogenic mediator independent of CB receptors, but in higher doses, it induced apoptosis of HSCs, which may have antifibrotic properties [18]. The aforementioned study by Julien et al. demonstrated that 2-AG showed growth inhibitory and cytotoxic effects independent of CB2 [25]. It also showed that the exocannabinoid, THC, causes apoptosis by oxidative stress [25]. In particular, THC suppresses proliferation of hepatic myofibroblasts and stellate cells and induces their apoptosis, which may reveal antifibrotic, hepatoprotective mechanisms [18].

The endocannabinoid system, in addition to mediating effects on cardiovascular complications of cirrhosis, may be a target for treatment in hepatic encephalopathy secondary to decompensated cirrhosis. CB1 blockage by rimonabant and activation of CB2 by HU-308 demonstrated improved neurologic score and cognitive function in mice with hepatic encephalopathy [19,24,30]. 2-AG was found to improve hepatic encephalopathy [19,30]. Cannabidiol, independent of CB1 and CB2, was found to improve cognitive and motor function, as well as neuroinflammation in patients with hepatic encephalopathy [19].

Specific findings regarding the role and impact of the endocannabinoid system have been studied for etiologies of chronic liver disease that can progress to cirrhosis. In those with hepatitis C, daily cannabis use was associated with more severe fibrosis and steatosis [34]. Dai et al. demonstrated that CB1 and CB2 were expressed in patients with chronic hepatitis B and the degree of fibrosis was increased with increased expression of both [35]. In alcoholic liver disease, animal studies demonstrated that blockade of CB2 results in more pronounced liver damage after ethanol intake, suggesting a protective mechanism for CB2 in the setting of chronic alcohol use [36]. In the same study, fibrogenesis was increased in CB1 blockade in the setting of chronic alcohol use [36]. In hepatic ischemic-reperfusion injury, the data regarding endocannabinoids are conflicting [22].

The roles of exocannabinoids are as aforementioned. As THC, functions on multiple receptors in addition to CB1 and CB2 [19], THCV was found as dose-dependent, functioning as a CB1 antagonist in low doses or a CB1 agonist in high doses [21,37]. In a study by Bolognini, et al., THCV was investigated for its anti-inflammatory properties particularly for its function as a CB1 antagonist in vivo and a CB2 agonist in vitro in humans and in vivo and vitro in mice [38]. CBD functions primarily on CB1 [21]. Therefore, based on the associations made with CB1 and CB2 agonism and antagonism in the literature, hypotheses can be developed as to the effect of THC, cannabidiol, THCV, and cannabis in cirrhosis although prospective studies are warranted.

## 11. Conclusions

The socioeconomic burden and high mortality rates associated with cirrhosis emphasize the need for therapeutic interventions. Because most treatment recommendations for cirrhosis aim to address complications of decompensated cirrhosis, there is a need for further investigation of antifibrogenic therapies that can slow the progression of fibrosis and thereby prevent cirrhosis and its complications. The endocannabinoid system has demonstrated powerful antifibrogenic as well as profibrogenic properties, as summarized in Table 1 and Table 2, that can be pharmacologically manipulated to potentially treat cirrhosis at the histologic level. Further research is warranted to develop pharmacotherapies that utilize this endogenous system as a means of treating a debilitating, costly disease. Additionally, further studies investigating exocannabinoid use in cirrhosis may be warranted as marijuana is the most widely used illicit drug in the United States to recommend for or against its use in this context [39].

## Figures and Tables

**Table 1:** Functions of Cannabinoid Receptors of the Endocannabinoid System in Cirrhosis.

**CB1 Agonism**	Causes fibrogenesis (within in vitro studies) [17] Possibly increases liver regeneration (in rodent studies) [29]
**CB1 Antagonism/Deficiency**	Improves hepatic fibrosis and steatosis (in rodent studies) [19,22,26] Decreases TGF-beta (in rodent studies) [23,24] Decreases activation of HSCs (in rodent studies) [23,24] Associated with decreased collagen deposition in cirrhotic livers (in rodent studies) [28] Reduces liver regeneration (in rodent studies) [29] Increases arterial pressure and vascular resistance with reduced mesenteric blood flow and portal pressure (in animal studies) [19], preventing ascites (in animal studies) [24,31,32] Improves neurologic score and cognitive function in hepatic encephalopathy (in human studies) [19,24,30] Increases apoptosis of activated myofibroblasts (in rodent studies) [23,24]
**CB2 Agonism**	Conteracts progression to fibrosis (within in vitro studies) [17,25] Prevention of fibrosis of pregession (in rodent studies) [24,26] Accelerates regenerative response following acute liver injury (in rodent studies) [17] Triggers growth inhibition and apoptosis (within in vitro studies) [25] Enhances regenerative response to acute liver injury (in rodent studies) [18] Improves liver fibrosis (in rodent studies) [27] Accelerates liver regeneration (in rodent studies) [18] Decreases inflammatory infiltrate (in rodent studies) [27] Improves neurologic score and cognitive function in hepatic encephalopathy (in rodent studies) [24,31,32] Associated with decreased density of hepatic myofibroblasts secondary to apoptosis (in rodent studies) [27]
**CB2 Antagonism/Deficiency**	Increased collagen deposition, steatosis, and enhanced inflammation (in rodent studies) [19,22] Prolongs survival of liver fibrogenic cells, increasing fibrosis (in rodent studies) [24,25]

**Table 2:** Functions of Endocannabinoids and Exocannabinoids in Cirrhosis.

**AEA**	Antifibrogenic; inhibits proliferation of HSC and induces necrotic death [30] Induces mesenteric vasodilation and hypotension via CB1 induction (in rodent studies) [18,19] Contributes to cirrhotic cardiomyopathy (in rodent studies) [24] Induces hepatocyte proliferation (in animal studies) [19]
**2-AG**	Fibrogenic mediator (in rodent studies) [18] Growth inhibitory and cytotoxic effects (in rodent studies) [18] In higher doses, induces apoptosis of HSCs, (antifibrogenic) (in rodent studies) [18] Improves hepatic encephalopathy (in rodent studies) [24,30]
**THC**	Suppresses proliferation of hepatic myofibroblasts and stellate cells (in rodent studies) [18] Induces apoptosis (in rodent studies) [18]
**Cannabidiol**	Improves cognitive, motor function, and neuroinflammation in hepatic encephalopathy (in human studies) [19]

## References

[1] Heidelbaugh J.J., Bruderly M. (2006). Cirrhosis and chronic liver failure: Part I. Diagnosis and evaluation. Am. Fam. Physician.

[2] Neff G.W., Duncan C.W., Schiff E.R. (2011). The Current Economic Burden of Cirrhosis. Gastroenterol. Hepatol..

[3] Scaglione S., Kliethermes S., Cao G., Shoham D., Durazo R., Luke A., Volk M.L. (2015). The epidemiology of cirrhosis in the United States: A population-based study. J. Clin. Gastroenterol..

[4] Byass P. (2014). The global burden of liver disease: A challenge for methods and for public health. BMC Med..

[5] Sharma S., Khalili K., Nguyen G.C. (2014). Non-invasive diagnosis of advanced fibrosis and cirrhosis. World J. Gastroenterol..

[6] Centers for Disease Control and Prevention Chronic Liver Disease and Cirrhosis. http://www.cdc.gov/nchs/fastats/liver-disease.htm.

[7] Elpek G.Ö. (2014). Cellular and molecular mechanisms in the pathogenesis of liver fibrosis: An update. World J. Gastroenterol..

[8] Gressner A.M. (1998). The cell biology of liver fibrogenesis—An imbalance of proliferation, growth arrest and apoptosis of myofibroblasts. Cell Tissue Res..

[9] Zhou W.-C., Zhang Q.-B., Qiao L. (2014). Pathogenesis of liver cirrhosis. World J. Gastroenterol..

[10] Friedman S.L. (2013). Pathogenesis of Hepatic Fibrosis.

[11] Rockey D.C., Caldwell S.H., Goodman Z.D., Nelson R.C., Smith A.D. (2009). Liver biopsy. Hepatology.

[12] Garcia-Tsao G., Abraldes J.G., Berzigotti A., Bosch J. (2017). Portal hypertensive bleeding in cirrhosis: Risk stratification, diagnosis, and management: 2016 practice guidance by the American Association for the study of liver diseases. Hepatology.

[13] Thornton K. Lesson 5. Evaluation and Prognosis of Patients with Cirrhosis. https://www.hepatitisc.uw.edu/go/evaluation-staging-monitoring/evaluation-prognosis-cirrhosis/core-concept/all.

[14] Heidelbaugh J.J., Sherbondy M. (2006). Cirrhosis and chronic liver failure: Part II. Complications and treatment. Am. Fam. Physician.

[15] Runyon B.A. (2013). Introduction to the revised American Association for the Study of Liver Diseases Practice Guideline management of adult patients with ascites due to cirrhosis 2012. Hepatology.

[16] European Association for the Study of the Liver (2010). EASL clinical practice guidelines on the management of ascites, spontaneous bacterial peritonitis, and hepatorenal syndrome in cirrhosis. J. Hepatol..

[17] Mallat A., Teixeira-Clerc F., Deveaux V., Manin S., Lotersztajn S. (2011). The endocannabinoid system as a key mediator during liver diseases: New insights and therapeutic openings. Br. J. Pharmacol..

[18] Tam J., Liu J., Mukhopadhyay B., Cinar R., Godlewski G., Kunos G. (2011). Endocannabinoids in liver disease. Hepatology.

[19] Patsenker E., Stickel F. (2016). Cannabinoids in liver diseases. Clin. Liver Dis..

[20] Silvestri C., Paris D., Martella A., Melck D., Guadagnino I., Cawthorne M., Motta A., Di Marzo V. (2015). Two non-psychoactive cannabinoids reduce intracellular lipid levels and inhibit hepatosteatosis. J. Hepatol..

[21] Jadoon K.A., Ratcliffe S.H., Barrett D.A., Thomas E.L., Stott C., Bell J.D., O’Sullivan S.E., Tan G.D. (2016). Efficacy and safety of cannabidiol and tetrahydrocannabivarin on glycemic and lipid parameters in patients with type 2 diabetes: A randomized, double-blind, placebo-controlled, parallel group pilot study. Diabetes Care.

[22] Pacher P., Gao B. (2008). Endocannabinoids and Liver Disease. III. Endocannabinoid effects on immune cells: Implications for inflammatory liver diseases. Am. J. Physiol. Gastrointest. Liver Physiol..

[23] Teixeira-Clerc F., Julien B., Grenard P., Van Nhieu J.T., Deveaux V., Li L., Serriere-Lanneau V., Ledent C., Mallat A., Lotersztajn S. (2006). CB1 cannabinoid receptor antagonism: A new strategy for the treatment of liver fibrosis. Nat. Med..

[24] Baldassarre M., Giannone F.A., Napoli L., Tovoli A., Ricci C.S., Tufoni M., Caraceni P. (2013). The endocannabinoid system in advanced liver cirrhosis: Pathophysiological implication and future perspectives. Liver Int..

[25] Julien B., Grenard P., Teixeira-Clerc F., Van Nhieu J.T., Li L., Karsak M., Zimmer A., Mallat A., Lotersztajn S. (2005). Antifibrogenic role of the cannabinoid receptor CB2 in the liver. Gastroenterology.

[26] Mallat A., Lotersztajn S. (2006). Endocannabinoids as novel mediators of liver diseases. J. Endocrinol. Investig..

[27] Muñoz-Luque J., Ros J., Fernández-Varo G., Tugues S., Morales-Ruiz M., Alvarez C.E., Friedman S.L., Arroyo V., Jiménez W. (2008). Regression of fibrosis after chronic stimulation of cannabinoid CB2 receptor in cirrhotic rats. J. Pharmacol. Exp. Ther..

[28] Yang Y.Y., Lin H.C., Huang Y.T., Lee T.Y., Hou M.C., Wang Y.W., Lee F.Y., Lee S.D. (2007). Effect of chronic CB1 cannabinoid receptor antagonism on livers of rats with biliary cirrhosis. Clin. Sci..

[29] Mukhopadhyay B., Cinar R., Yin S., Liu J., Tam J., Godlewski G., Harvey-White J., Mordi I., Cravatt B.F., Lotersztajn S. (2011). Hyperactivation of anandamide synthesis and regulation of cell-cycle progression via cannabinoid type 1 (CB1) receptors in the regenerating liver. Proc. Natl. Acad. Sci. USA.

[30] Parfieniuk A., Flisiak R. (2008). Role of cannabinoids in chronic liver diseases. World J. Gastroenterol..

[31] Mallat A., Teixeira-Clerc F., Lotersztajn S. (2013). Cannabinoid signaling and liver therapeutics. J. Hepatol..

[32] Domenicali M., Caraceni P., Giannone F., Pertosa A.M., Principe A., Zambruni A., Trevisani F., Croci T., Bernardi M. (2009). Cannabinoid type 1 receptor antagonism delays ascites formation in rats with cirrhosis. Gastroenterology.

[33] Gabbay E., Avraham Y., Ilan Y., Israeli E., Berry E.M. (2005). Endocannabinoids and liver disease—Review. Liver Int..

[34] Hézode C., Zafrani E.S., Roudot–Thoraval F., Costentin C., Hessami A., Bouvier–Alias M., Medkour F., Pawlostky J.M., Lotersztajn S., Mallat A. (2008). Daily cannabis use: A novel risk factor of steatosis severity in patients with chronic hepatitis C. Gastroenterology.

[35] Dai E., Zhang L., Ye L., Wan S., Feng L., Qi Q., Yao F., Li Z. (2017). Hepatic expression of cannabinoid receptors CB1 and CB2 correlate with fibrogenesis in patients with chronic hepatitis B. Int. J. Infect. Dis..

[36] Trebicka J., Racz I., Siegmund S.V., Cara E., Granzow M., Schierwagen R., Klein S., Wojtalla A., Hennenberg M., Huss S. (2011). Role of cannabinoid receptors in alcoholic hepatic injury: Steatosis and fibrogenesis are increased in CB2 receptor-deficient mice and decreased in CB1 receptor knockouts. Liver Int..

[37] Pertwee R.G. (2008). The diverse CB1 and CB2 receptor pharmacology of three plant cannabinoids: Δ9-tetrahydrocannabinol, cannabidiol and Δ9-tetrahydrocannabivarin. Br. J. Pharmacol..

[38] Bolognini D., Costa B., Maione S., Comelli F., Marini P., Di Marzo V., Parolaro D., Ross R.A., Gauson L.A., Cascio M.G. (2010). The plant cannabinoid Δ9-tetrahydrocannabivarin can decrease signs of inflammation and inflammatory pain in mice. Br. J. Pharmacol..

[39] Bose J., Hedden S.L., Lipari R.N., Park-Lee E., Porter J.D., Pemberton M.R. (2016). Key Substance Use and Mental Health Indicators in the United States: Results from the 2015 National Survey on Drug Use and Health.

